# Critical pharmacokinetic and pharmacodynamic drug-herb interactions in rats between warfarin and pomegranate peel or guava leaves extracts

**DOI:** 10.1186/s12906-019-2436-5

**Published:** 2019-01-24

**Authors:** Maisa Alnaqeeb, Kenza A. Mansor, Eyad M. Mallah, Bayan Y. Ghanim, Nasir Idkaidek, Nidal A. Qinna

**Affiliations:** 1 Department of Pharmacology and Biomedical Sciences, Faculty of Pharmacy and Medical Sciences, University of Petra, Airport Road, P.O. Box 961343, Amman, Jordan; 2 Department of Pharmaceutical Medicinal Chemistry and Pharmacognosy, Faculty of Pharmacy and Medical Sciences, University of Petra, Amman, Jordan; 3 University of Petra Pharmaceutical Center (UPPC), University of Petra, Amman, Jordan

**Keywords:** Drug-herb interactions, Bleeding, Pomegranate peel, Ellagic acid, Guava leaves, Cytochrome P450

## Abstract

**Background:**

In-depth information of potential drug-herb interactions between warfarin and herbal compounds with suspected anticoagulant blood thinning effects is needed to raise caution of concomitant administration. The current study aimed to investigate the impact of co-administration of pomegranate peel and guava leaves extracts, including their quality markers namely; ellagic acid and quercetin, respectively, on warfarin’s in vivo dynamic activity and pharmacokinetic actions, in addition to potential in vitro cytochrome P450 enzymes (CYP) inhibition.

**Methods:**

Influence of mentioned extracts and their key constituents on warfarin pharmacodynamic and kinetic actions and CYP activity were evaluated**.** The pharmacodynamic interactions were studied in Sprague Dawley rats through prothrombin time (PT) and International Normalized Ratio (INR) measurements, while pharmacokinetic interactions were detected in vivo using a validated HPLC method. Furthermore, potential involvement in CYP inhibition was also investigated in vitro on isolated primary rat hepatocytes.

**Results:**

Preparations of pomegranate peel guava leaf extract, ellagic acid and quercetin in combination with warfarin were found to exert further significant increase on PT and INR values (*p* < 0.01) than when used alone (*p* < 0.05). Pomegranate peel extract showed insignificant effects on warfarin pharmacokinetics (*p > 0.05*), however, its constituent, namely, ellagic acid significantly increased warfarin C_max_ (*p < 0.05*). Guava leaves extract and quercetin resulted in significant increase in warfarin C_max_ when compared to control (*p < 0.01*). Furthermore, guava leaves extract showed a significant effect on changing the AUC, CL and V_z_. Significant reduction in CYP2C8, 2C9, and 3A4 was seen upon concomitant use of warfarin with ellagic acid, guava leaves and quercetin, unlike pomegranate that insignificantly affected CYP activities.

**Conclusion:**

All combinations enhanced the anticoagulant activity of warfarin as the results of in vivo and in vitro studies were consistent. The current investigation confirmed serious drug herb interactions between warfarin and pomegranate peel or guava leaf extracts. Such results might conclude a high risk of bleeding from the co-administration of the investigated herbal drugs with warfarin therapy. In addition, the results raise attention to the blood-thinning effects of pomegranate peel and guava leaves when used alone.

## Background

The wide spread of using natural products among people raised interest in their therapeutic activity as an alternative medicine. Furthermore, the complex mixture of bioactive constituents makes it important to evaluate potential interactions between natural products and prescribed drugs, especially, when used concomitantly with drugs that have narrow therapeutic index.

Many studies were published evaluating the potential metabolic or pharmacologic interactions between natural products and warfarin. An example of the former includes alteration of vitamin K bioavailability through some herbs that might inhibit synthesis of vitamin K by intestinal flora, like thyme and garlic which influence vitamin K-dependent clotting factor synthesis, thereby enhance the effect of warfarin [1]. The most important kinetic interactions are those interfering with warfarin hepatic metabolism. For example, St John’s wort induces microsomal cytochrome P450 (CYP) enzyme-mediated metabolism of warfarin, particularly CYP2C9 and increases warfarin clearance [2].

The contribution of bioactive phytochemicals including polyphenols, phytosterols has been raising concern in CYP-associated metabolic studies for potential drug-herb interactions, as the consumption and use of herbal supplements increased in the recent years. Many studies reported the involvement of flavonoids including quercetin, ellagitannins and others in triggering CYP450 enzyme activities [3–6], therefore they have been concerned in the current study. Pomegranate peel (*Punica granatum* L., Lythraceae) and guava leaves (*Psidium guava* L., Myrtaceae) extracts are examples of widely used natural products due to their pharmacological actions. Many studies demonstrated that pomegranate peel has anti-diarrheal, anti-ulcerative, anti-oxidant and anti-inflammatory effects [7–10]. Pomegranate peel is an important source of many bioactive compounds including phenolic compounds like punicalagins, flavonoids like quercetin (Que), ellagitannins such as ellagic acid (EA) and gallic acid [11]. It has been reported that pomegranate peel could possess in vitro thrombolytic activity when added to clots formed from human blood samples, extracts of it were reported to cause prolonged bleeding and thrombin time [12]. In addition to its reported ability to alter Prothrombin time (PT), reduce platelet aggregation and protect against hematotoxicty [13–16].

Guava leaves possess anti-tussive, anti-hypertensive, anti-hyperglycemic and anti-hyperlipidemic activities [17–19]. Flavonoids including quercetin and its glycoside are major constituents in guava leaves extract [20]. Anticoagulant properties of guava leaves through its major phytochemicals including gallic acid and quercetin have been also reported [21]. In fact, the presence of quercetin in guava leaves raise the concern of inducing drug-herb interaction between guava and warfarin since quercetin and its metabolites were reported to induce antiplatelet and anticoagulant activities in additon to its interaction of displacing warfarin from its protein binding site [22–24].

Owing to the capacity of such phytochemical compounds in altering the parameters mentioned earlier, this study aimed to investigate the impact of concomitant administration of pomegranate peel and guava leaves extracts, including their quality markers; ellagic acid and quercetin, respectively, on warfarin activity and bioavailability pre clinically in Sprague Dawley rats as a suitable model for warfarin’s action. In addition, the potential inhibition of selected CYP enzymes that are responsible for warfarin metabolism is investigated with the herbal drugs in vitro on rat isolated primary hepatocytes.

## Methods

### Materials

Warfarin, quercetin hydrate and ellagic acid were purchased from Sigma-Aldrich (Missouri, USA). Standardized guava leaves extract (*Psidium guava* L.) (5% quercetin) and pomegranate peel extract (*Punica granatum* L.) (40% ellagic acid) were obtained from Gehrlicher GmbH (Eurasburg, Germany), and Puritans Pride (California, USA), respectively.

Carboxy Methyl Cellulose (CMC) was obtained from SciChem (West Midlands, England). For PT test, Néoplastine® CI Plus kit (Stago S.A.S, France) was used and tested on semi-automated, dual-channel coagulometer BFT II analyzer (Siemens GmbH, Erlangen, Germany). Tri-sodium citrate 3.2% (0.109 mol/L) and EDTA micro tubes were used to collect blood samples. As for HPLC analysis, deionized water (Nanopur™), methanol and acetonitrile (Chromanorm®) were obtained from Fisher Scientific Ltd. (Loughborough, UK). Phosphoric acid and triethylamine were purchased from Tedia (California, USA). As for metronidazole benzoate, it was a kind gift from the Jordanian Pharmaceutical Manufacturing Company (Amman, Jordan).

For the isolation of rat primary hepatocytes; Hank’s Balanced Salt Solution (HBSS) (Ca^2+^ and Mg^2+^ free) and HBSS (with Ca^2+^, and Mg^2+^) were obtained from Invitrogen (California, USA). Dulbecco’s Modified Eagle Medium (DMEM) was purchased from EuroClone (Pero, Italy). 4-(2-hydroxyethyl)-1-piperazine-ethanesulfonic acid (HEPES) was obtained from Alfa Aesar (Heysham, England). Fetal bovine serum (FBS) and penicillin-streptomycin solution were obtained from Biowest (California, USA) and Caisson Lab (Rhode Island, USA), respectively. Collagenases II was purchased from Gibco BRL (Maryland, USA). Dimethyl sulfoxide (DMSO) was obtained from Scharlan Chemie S.A. (Barcelona, Spain). Sulfamethoxazole and trimethoprim were kindly provided by Hikma Pharmaceuticals Company (Amman, Jordan), whereas ketoconazole was kindly donated by Dar Al-Dawa Pharmaceuticals Company (Naur, Jordan). P450-Glo™ CYP2C9 with Luciferin-H, P450-Glo™ 2C8 with Luciferin-ME and P450-Glo™ 3A4 with Luciferin-IPA Assay kits were purchased from Promega (Madison, USA).

### In vivo warfarin-herb interactions

CMC at a concentration of 0.5% (*w*/*v*) solution was prepared by dissolving it in water. Concentrations of warfarin solution, guava leaves extract, quercetin, pomegranate peel extract and ellagic acid suspensions were calculated per content concentrations provided by the manufacturers to obtain the needed corresponding doses. Treatments were freshly prepared by dissolving an accurately weighed amount of each material in 0.5% CMC solution.

#### In vivo pharmacodynamic study design

Adult Sprague Dawley laboratory rats were supplied by the Animal Facility of the University of Petra (Amman, Jordan). Healthy rats were housed under controlled conditions including humidity between 55 to 65%, temperatures between 22 to 24 °C and photoperiod cycles of 12 h light/12 h dark in conventional polycarbonate cages of suitable size. Rats were fasted overnight with free access to water. All experiments involving animals were conducted according to the institutional guidelines for the use of laboratory animals, which follows the guidelines of the Federation of European Laboratory Animal Science Associations (FELASA). The used animals were euthanized with cervical dislocation at the end of each experiment. The study protocol was revised and approved by the Committee of Scientific Research of the University of Petra (Approval number: 4–2016/2017, Date: 22/06/2017). The rats were weighed and randomized into ten groups (*n* = 8) and acclimatized for ten days before the experiment. Oral stainless steel gavage needle (Harvard Apparatus, Kent, UK) was used for oral administration of the treatments. Groups were treated daily for five days as following; group 1 received oral 0.5% CMC solution and served as a negative control. As for group 2, 0.5 mg/kg warfarin solution was daily administered as a positive control [25]. Group 3 received 100 mg/kg pomegranate peel extract suspension [26]. While group 4 received warfarin orally in combination with pomegranate peel extract suspension. Group 5 received doses of 40 mg/kg ellagic acid which represents 40% of pomegranate peel extract [27, 28], as group 6 received a combination of warfarin and ellagic acid. Group 7 received 250 mg/kg guava leaves extract suspension [29]. While group 8 received warfarin in combination with guava leaves extract suspension. Finally, groups 9 and 10, received 12.75 mg/kg quercetin, which represents 5.1% of guava leaf extract, and a combination of warfarin and quercetin suspensions, respectively.

#### Sample collection

On the fifth day, blood samples were collected from rats and drawn into sodium citrate (3.2%) micro tubes. The micro tubes were centrifuged at 7000 RPM for 10 min. Plasma was separated, transferred directly into labeled Eppendorf tubes for PT and INR analysis.

#### Measurement of PT and INR

Reconstituted thromboplastin was added to 100 μl of each plasma sample in pre-warmed cuvette at 37 °C. After 60 s, 200 μl of starting reagent was added and PT was recorded. INR was calculated from the detected PT according to the manufacturer’s instructions.

#### In vivo pharmacokinetic parallel study design

Sprague-Dawley rats with an average weight of 220 ± 10 g were used to study the effect of natural products (pomegranate peel and guava leaves extracts) in addition to their quality markers (ellagic acid and quercetin) on warfarin pharmacokinetics. Rats were randomized into five groups (*n* = 8) where a control group received a single dose of 0.5 mg/kg warfarin solution on the day of experiment. The other four groups received a daily single dose of 250 mg/kg guava leaves extract, 100 mg/kg pomegranate peel, 12.75 mg/kg quercetin and 40 mg/kg ellagic acid for five days in combination with a single oral dose of 0.5 mg/kg warfarin administered on the fifth day [25–29].

#### Sample collection

On the fifth day, blood samples were collected from the 18 h fasted rats at different time intervals namely; at zero time, 0.5, 1, 2, 3, 4, 6, 8, 12, 24, 48 and 72 h from all groups and drawn into EDTA micro tubes. The micro tubes were centrifuged at 7000 RPM for 10 min. Plasma was separated, transferred directly into labeled Eppendorf tubes and stored at − 20 °C until requested for HPLC analysis.

#### Chromatographic conditions

**Fig. 1:**
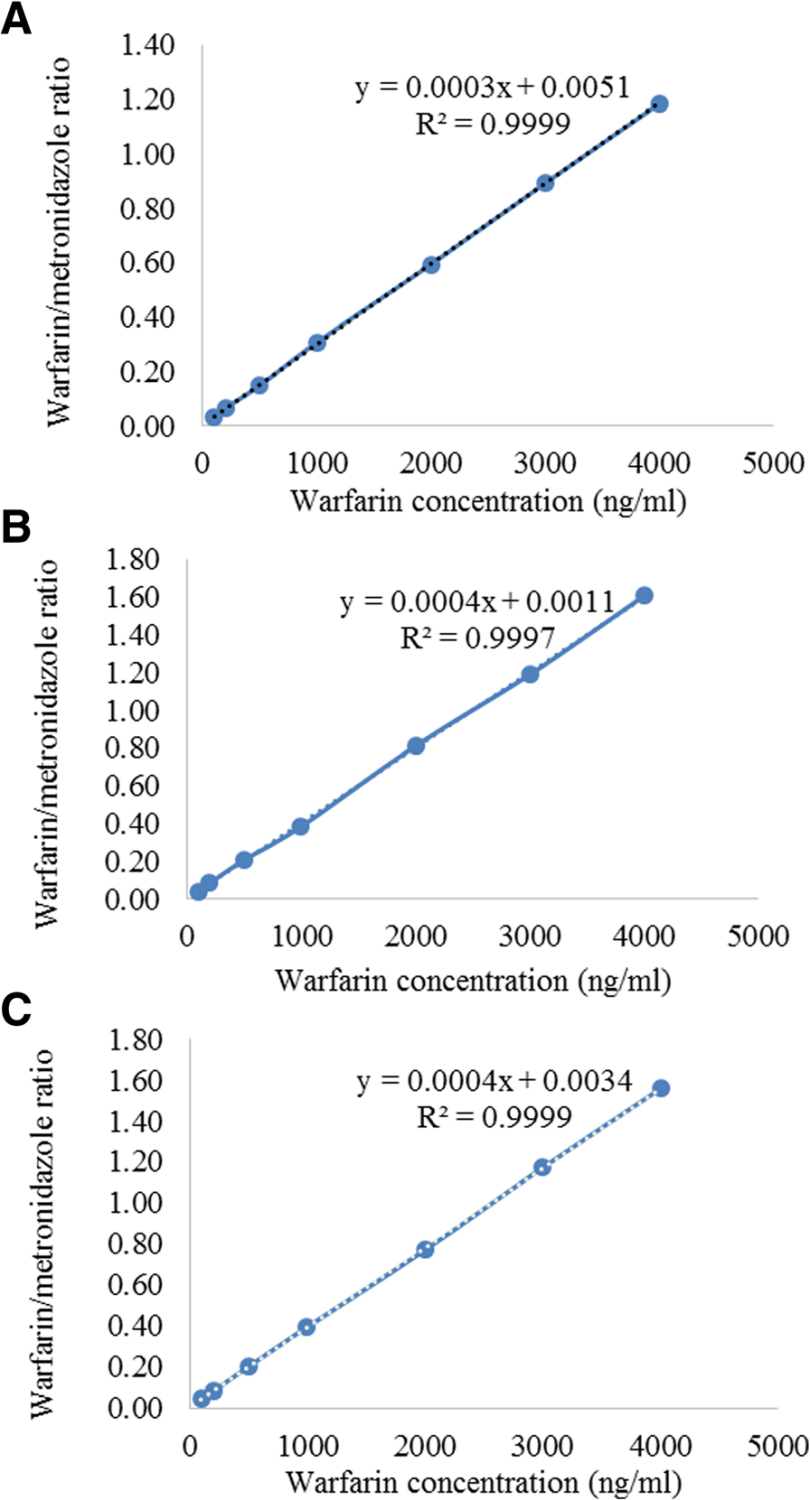
Calibration curves of warfarin HPLC analysis at (**a**) day 1 (**b**) day 2 (**c**) day 3

Reversed-phase high-performance liquid chromatography (HPLC) analysis was used to separate samples using Finnigan surveyor system (Thermo Electron Corporation, San Jose, California, USA) on a hypersil™ BDS C-18 Column (150 mm × 4.6 mm, 5 μm) (Thermo Electron Corporation, San Jose, California, USA) and quantified using ultraviolet detection at 310 nm (UV-VIS Plus Detector). The mobile phase was a mixture of 60% acetonitrile HPLC grade and 40% water containing 1 ml triethylamine/l and phosphoric acid (pH 3.0) with a flow rate of 1.0 ml/min (LC pump plus) using 20 μl injection volume (Autosampler Plus). Metronidazole benzoate was used as an internal standard. ChromQuest 4.2.34 chromatography data system was implemented for data acquisition and handling. Calibration curve and quality control (QC) samples were prepared by analyzing different concentrations obtained by diluting warfarin stock solution (400 μg/ml) (Fig. 1, Table 1). Consequently, different concentrations of working solutions were prepared for plasma spiking by mixing 25 μl of working solutions in 975 μl of plasma (Fig. 1, Table 1). To extract warfarin from test samples, aliquots of samples (100 μl) were mixed with 150 μl of acetonitrile containing metronidazole benzoate (I.S., 1.5 μg/ml) and centrifuged at 14000 RPM for 10 min. The supernatant was then analyzed using an in house validated HPLC method at the Faculty of Pharmacy and Medical Sciences, University of Petra, Amman, Jordan (MSc thesis, Farah Al-Mamoori, 2016, University of Petra). In brief, the analysis method was subjected to full validation where the accuracy, precision, linearity recovery and stability were all calculated and found to be within acceptance criteria of validation guidelines. Inter-day precision and accuracy with CV% range of 1.22–4.85% and accuracy range of 97.95–107.76% were obtained. The coefficient of correlation was 0.9991 with reasonable sensitivity and selectivity.

**Table 1:** Accuracy percentage based on the measured warfarin concentrations of standard points and QC samples. The mean measurements are for day 1, day 2 and day 3

Final calculated concentration (ng/ml)	Mean measured concentration (ng/ml)	Standard Deviation (SD)	Coefficient of Variation (%CV)	Accuracy (%)
100	96.84	2.19	2.27	96.84
200	201.61	9.15	4.54	100.8
500	495.13	19.45	3.93	99.03
1000	981.22	23.39	2.38	98.12
2000	1969.12	53.24	2.71	98.46
3000	2950.57	23.15	0.78	98.35
4000	3945.3	63.52	1.61	98.63
QC low (300)	300.28	12.47	4.15	100.09
QC mid (1800)	1747.26	28.48	1.63	97.07
QC high (3500)	3493.65	239.92	6.87	99.82

#### Pharmacokinetic parameters calculations

Noncompartmental analysis was used to calculate the detected individual pharmacokinetic parameters for warfarin in the in vivo plasma samples using the Winnonlin® program (version 5.2). The calculated pharmacokinetic parameters presented in the current study were maximum plasma concentration (C_max_), area under the curve (AUC), area under the first moment curve (AUMC), mean residence time (MRT), half-life (t_0.5_), clearance (CL), volume of distribution (V_z_), time at which C_max_ was observed (T_max_) and elimination rate constant (K_el_).

### In vitro cytochrome determinations on primary isolated rat hepatocytes

#### Preparation of buffers

All perfusion buffers were freshly prepared under sterile conditions and were warmed for 30 min in a water bath (Elmasonic S, Elma, Germany) at 40 °C. HBSS, without Ca^2+^ and Mg^2+^ was used as a perfusion buffer I. Perfusion buffer II was prepared by adding 1000 U of collagenase II to HBSS (with Ca^2+^ and Mg^2+^). This buffer was kept warm in a water-bath and used within 30 min after preparation. DMEM medium was prepared by the addition of 5% FBS, 100 IU/ml penicillin and 100 mg/ml of streptomycin to DMEM medium.

#### Liver isolation and incubation

The liver of anaesthetized rat with inhaled isoflurane were perfused with collagenase enzyme as described by [30]. Liver cells were dispersed gently in a sterile petri dish, and prepared for hepatocyte culture as described elsewhere [31]. Cell were plated at a final concentration of 50,000 cell/100 μl and seeded in 96 well plates (100 μl). Cells were left to recover and grow overnight prior to the day of experiment.

#### Treatment of the cultured cells

After 16 h of incubation, each well of cultured rat hepatocytes were treated with 25 μl of warfarin, guava leaves extract, quercetin, pomegranate peel extract, ellagic acid, a combination of warfarin with each test compound in addition to the control CYP inhibitors namely; ketoconazole, sulfamethoxazole or trimethoprim. Doses of chemicals selected for in vitro testing on primary cultured hepatocyte mitochondrial activity were estimated after literature review and preliminary work in order to obtain the IC_50_ values of tested compounds (data not shown). All treatments were dissolved in DMSO then diluted with the DMEM medium to obtain a final concentrations of 3 μg/ml warfarin [32], 294 μg/ml guava leaves extract, 15 μg/ml quercetin [33, 34], 120 μg/ml pomegranate peel extract, 48 μg/ml ellagic acid, 1 μg/ml ketoconazole, 130 μg/ml sulfamethoxazole and 30 μg/ml trimethoprim. Directly after the addition of treatments, 25 μl of the lumenogenic substrate (Luciferin-H, Luciferin-ME or Luciferin-IPA) was added to each well. The plates were incubated at 37 °C for appropriate time according to manufacture protocol (Promega, USA). Later, 25 μl from culture media from each well was transferred into 96-well opaque white luminometer plate and equal amount of Luciferin detection reagent was added to each well to initiate the luminescent reaction. The reaction was allowed for 20 min at room temperature away from light. Finally, luminescence was read using GloMax®-Multi Detection System (Promega, USA).

### Statistical analysis

Statistical comparisons were made with Independent Student’s t-test using SPSS software (version 22). Each data point represents the mean ± the standard deviation (SD)*. P value* less than 0.05 was considered significant. The statistical analysis of the investigated pharmacokinetic parameters, was performed using ANOVA and Independent Student’s t-test after log transformation, except for T_max_ where Wilcoxon test was used.

## Results

### In vivo pharmacodynamics results

As illustrated in Table 2, both pomegranate peel and guava leaf extracts and their quality markers, ellagic acid and quercetin significantly increased PT and INR values upon concomitant administration with warfarin (*p < 0.01*). In addition, mentioned extracts and chemicals presented increase in PT and INR values when compared to control group (*p < 0.05).*

**Table 2:** Prothrombin time (PT) and International Normalized Ratio (INR) measurements of different rat groups treated with the natural products and their quality markers alone or in combination with warfarin

Treatment	Test	
	PT (s)	INR
Negative control	17.3 ± 0.53	1.0 ± 0.04
WF	191 ± 12	19 ± 1**
PPE	21.5 ± 1.2#	1.3 ± 0.09#
WF + PPE	360 ± 36.9**	42 ± 5.3**
EA	23 ± 1.31#	1.43 ± 0.10#
WF + EA	414 ± 139**	51 ± 19**
GvL	23.3 ± 1.04#	1.44 ± 0.08#
WF + GvL	425 ± 57**	52 ± 8.4**
QUE	22.5 ± 1.20#	1.39 ± 0.09#
WF + QUE	448 ± 79**	55 ± 11**

Each data point represents the mean ± SD (*n* = 8). **p < 0.01 (in comparison with warfarin alone). # *p < 0.01* (in comparison with the negative control)

### In vivo pharmacokinetics results

**Fig. 2:**
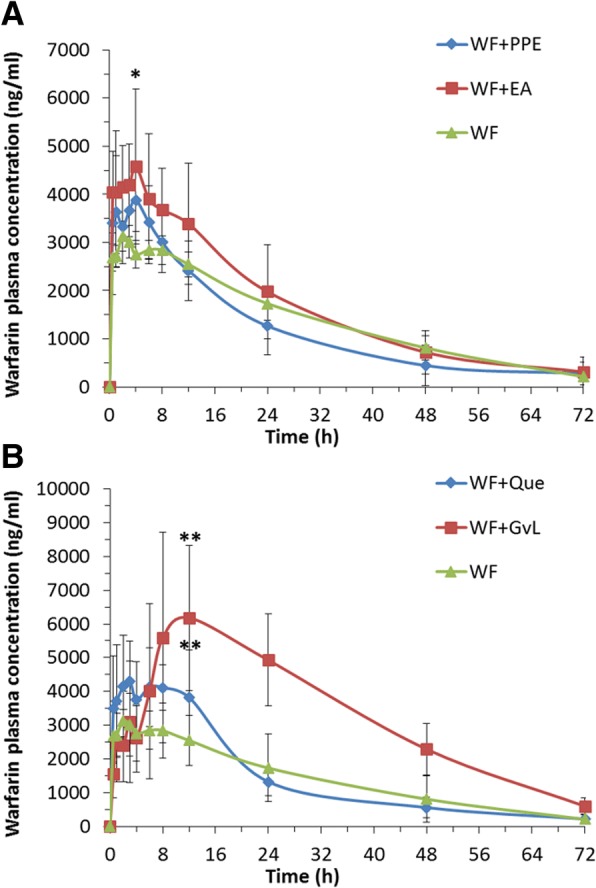
In vivo plasma concentrations of warfarin versus time curves in rats after a single oral dose of 0.5 mg/kg warfarin, along with warfarin in combination with: (**a**) five doses of pomegranate peel extract (100 mg/kg) and ellagic acid (40 mg/kg), (**b**) five doses of guava leaves extract (250 mg/kg) and quercetin (12.75 mg/kg). Each data point represents the mean ± SEM (*n* = 8). *p < 0.05, **p < 0.01

Pomegranate peel extract showed insignificant effects on warfarin pharmacokinetics (*p > 0.05*). Nonetheless, ellagic acid significantly increased warfarin C_max_ (*p < 0.05*) (Fig. 2a). As for pharmacokinetic interaction of warfarin with guava leaves extract and quercetin, the results showed that both treatments significantly increased warfarin C_max_ when compared to control (*p < 0.01*) (Fig. 2b). Furthermore, guava leaves extract showed a significant effect on changing the AUC, CL and V_z_ (Table 3).

**Table 3:** Pharmacokinetic parameters (PK) of warfarin alone (0.5 mg/kg) or in combination with the investigated natural products and their quality markers

PK Parameter	Treatment				
	WF (0.5 mg/kg)	WF & PPE (100 mg/kg)	WF & EA (40 mg/kg)	WF & GvL (250 mg/kg)	WF & Que. (12.75 mg/kg)
C_max_ (ng/ml)	3670 ± 663	4218 ± 843	4981 ± 1363*	6962 ± 2358**	5428 ± 1110**
T_max_ (h)	6 ± 8	3 ± 1.58	3 ± 1.58	11 ± 6	5 ± 4
AUC.inf (h*ng/ml)	106,613 ± 30,987	99,457 ± 43,116	131,661 ± 57,014	252,566 ± 51899**	115,904 ± 24,544
K_el_ (ml/h)	0.044 ± 0.01	0.035 ± 0.008	0.046 ± 0.01	0.044 ± 0.01	0.039 ± 0.006
t_0.5_ (h)	17 ± 4	21 ± 5	16 ± 4.6	17 ± 5	18 ± 3
MRT (h)	25 ± 5	25 ± 7.7	23 ± 6.5	30 ± 6	22 ± 6
CL/F (ml/h)	1.45 ± 0.5	1.54 ± 0.5	1.17 ± 0.4	0.61 ± 0.17**	1.14 ± 0.32
V_z_/F (ml)	35 ± 15	45 ± 11.4	26 ± 6.8	15 ± 5**	30 ± 8
AUMC-inf.(h*h*ng/m)	2,760,227 ± 1,140,965	2,762,909 ± 2,177,721	3,363,635 ± 2,416,054	7,630,361 ± 1894405**	2,594,574 ± 1,058,762
%Change in C_max_	–	15	36*	90**	48**
%Change in AUC.inf	–	− 7	23	137**	9
%Change in CL	–	6	−19	−58**	−21
%Change in V_z_	–	29	−26	−57**	−14

The data are presented as mean ± SD (*n* = 8). *p < 0.05, **p < 0.01

### In vitro cytochrome interactions

**Fig. 3:**
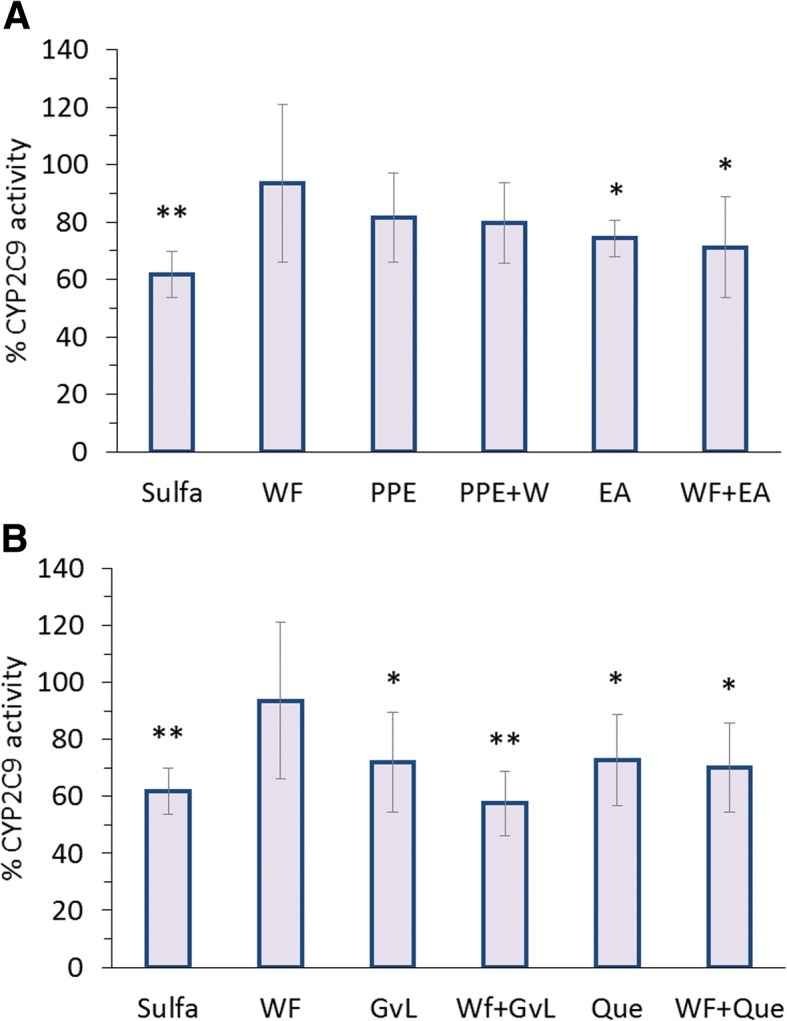
Percentage of CYP2C9 activity relative to untreated isolated hepatocytes of different groups treated with sulfamethoxazole (130 μg/ml) and warfarin (3 μg/ml) alone or in combination with: (**a**) pomegranate peel extract (120 μg/ml), ellagic acid (48 μg/ml) or, (**b**) guava leaves extract (294.1 μg/ml) and quercetin (15 μg/ml). Each data point represents the mean ± SD (*n* = 6). Statistical evaluation was performed by comparing the percentage of activity of untreated cells with treated cells *p < 0.05, **p < 0.01

**Fig. 4:**
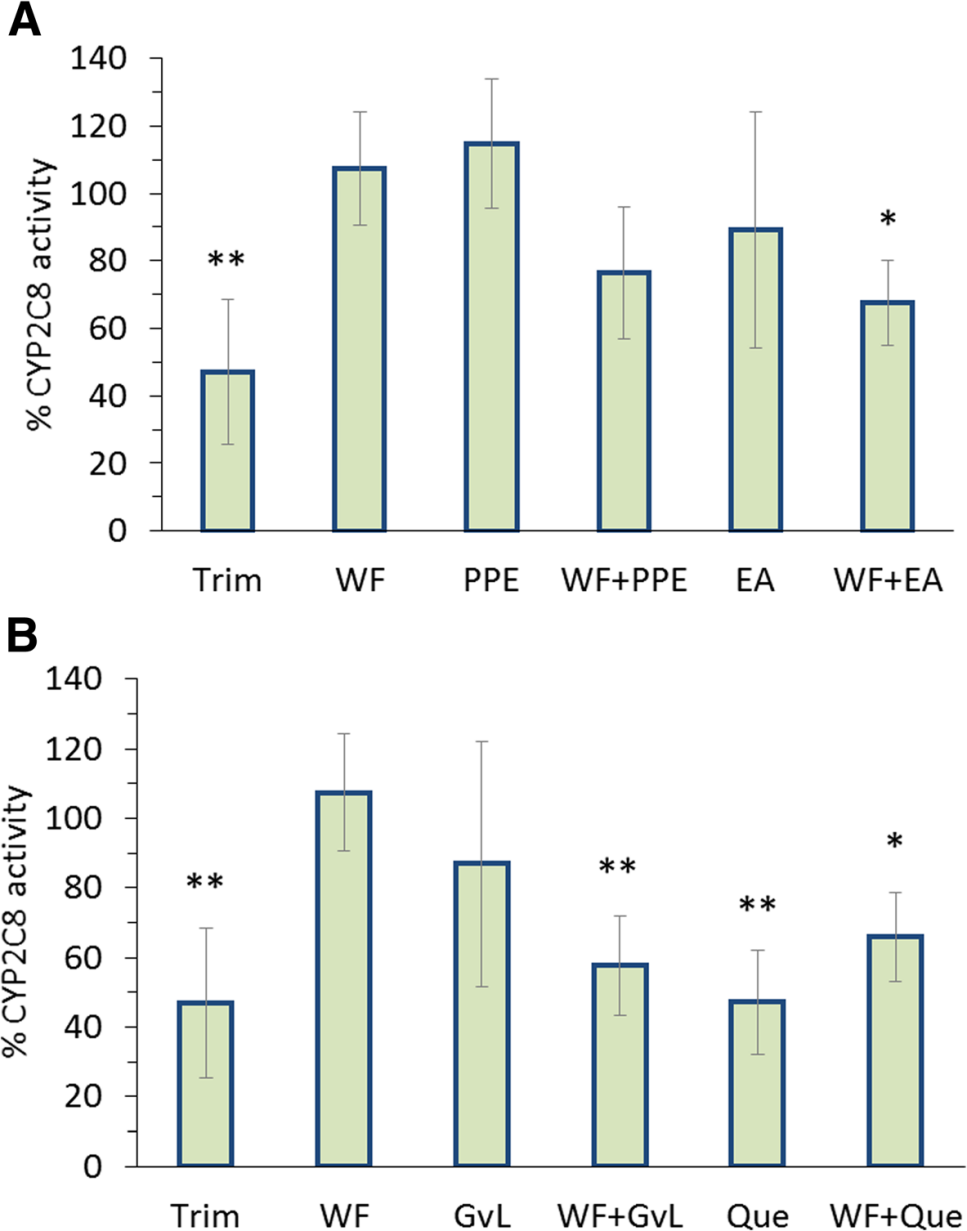
Percentage of CYP2C8 activity relative to untreated isolated hepatocytes of different groups treated with trimethoprim (30 μg/ml), and warfarin (3 μg/ml) alone or in combination with: (**a**) pomegranate peel extract (120 μg/ml), ellagic acid (48 μg/ml) or, (**b**) guava leaves extract (294.1 μg/ml) and quercetin (15 μg/ml). Each data point represents the mean ± SD (*n* = 6). Statistical evaluation was performed by comparing the percentage of activity of untreated cells with treated cells *p < 0.05, **p < 0.01

**Fig. 5:**
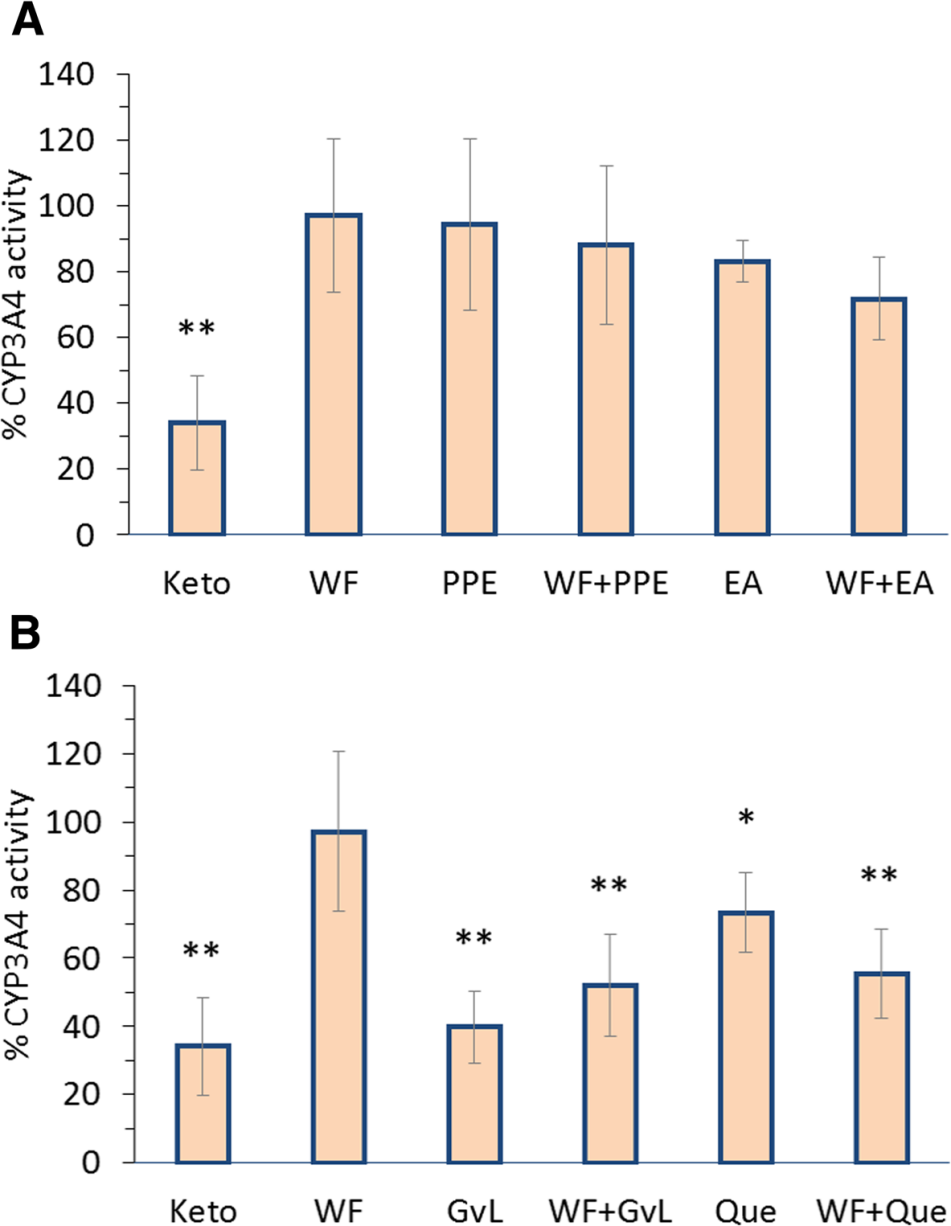
Percentage of CYP3A4 activity relative to untreated isolated hepatocytes of different groups treated with ketoconazole (1 μg/ml) and warfarin (3 μg/ml) alone or in combination with: (**a**) pomegranate peel extract (120 μg/ml), ellagic acid (48 μg/ml) or, (**b**) guava leaves extract (294.1 μg/ml) and quercetin (15 μg/ml). Each data point represents the mean ± SD (*n* = 6). Statistical evaluation was performed by comparing the percentage of activity of untreated cells with treated cells *p < 0.05, **p < 0.01

All utilized control inhibitors namely; sulfamethoxazole, trimethoprim and ketoconazole significantly reduced corresponding CYPs, CYP2C9, 2C8 and 3A4 activities, respectively (*p < 0.01*) (Figs. 3, 4 and 5). Activities of CYP2C9, 2C8 and 3A4 in primary cultured hepatocytes were not affected by warfarin (Figs. 3, 4 and 5). On the other hand, results indicated that pomegranate peel, whether used alone or in combination with warfarin, did not have any significant effect on all three CYPs (Figs. 3a, 4a and 5a). Combination of ellagic acid with warfarin significantly inhibited activities of CYP2C8, 2C9 and 3A4 (*p < 0.05*), however treatment of cells with ellagic acid alone presented significant inhibition on CYP2C9 activity despite other CYPs (*p < 0.05*) (Figs. 3a, 4a and 5a).

Although guava leaves extract showed an insignificant effect on CYP2C8 when used alone, it significantly reduced its activity when combined with warfarin (*p < 0.*01) (Fig. 4b) and however, inhibited the activities of CYP2C9 and 3A4 when used alone and concomitantly with warfarin (*p-values* are marked in Figs. 3b and 5b). Furthermore, quercetin inhibited activities of all three CYPs whether administered alone or concomitantly with warfarin (*p-values* are marked in Figs. 3b, 4b and 5b).

## Discussion

Significant drug-herb interactions were found between warfarin and the tested herbs. Pharmacodynamic results support a previous study reported by Sampath et al. (2016) which showed that pomegranate peel extract had in vitro thrombolytic activity when added to clots formed from human blood samples [13]. Besides, it was demonstrated that ellagitannins in pomegranate peel in addition to ellagic acid possessed strong action in reducing platelet aggregation when incubated with washed human platelet-rich plasma samples [14]. It was also reported that ellagic acid significantly inhibited human platelet aggregation against aggregation inducer in in vitro model [35]. Therefore, the increase in PT values post treating rats with pomegranate peel extract might be explained by the additive antiplatelet activity exerted by the extract. The impact of guava leaves and its quality marker, quercetin, on PT and INR values might be also attributed to the antiplatelet activity of flavonoids which are constituents of guava leaves extract, especially quercetin, which may potentially increase the risk of bleeding or potentiate the effects of warfarin therapy as previously reported [23]. An in vitro study showed that guava leaves extract along with some of its active constituents such as quercetin, gallic acid and ferulic acid had anticoagulant activity by preventing methylglyoxal-induced loss of activity of antithrombin III, thus, reduced glycation-associated hypercoagulable state that accelerate thrombosis formation [24]. No previous studies reported the effect of guava leaves extract and quercetin on the pharmacodynamics of warfarin. Therefore, the current investigation highlighted the presence of substantial interaction between warfarin and guava leaves and quercetin.

The investigated pharmacokinetic interactions of warfarin with pomegranate peel extract and ellagic acid confirmed that no significant effect was found in pomegranate peel extract on warfarin plasma concentration despite the increase in warfarin C_max_ upon ellagic acid administration. This could be explained by the low content of ellagitannins in pomegranate peel which consist of ellagic acid as a building block due to their large molecular size, poor lipid solubility and metabolism by intestinal flora [36, 37]. Noteworthy, the evaluation of CYP450 enzymes, namely, CYP2C9, 2C8, 3A4 was based on cell-based investigations and sufficient inhibition of corresponding well-known CYP control inhibitors including, sulfamethoxazole, trimethoprim and ketoconazole confirms that in vitro investigation of mentioned CYP isoforms on rat hepatocyte is possible, as raised in the literature [38, 39]. In regard to results presented for ellagic acid, and since it is known to exert inhibition on CYP2C9, its effect may be due to a decrease in hepatic first-pass metabolism of warfarin due to its ability to inhibit such CYP isoform (Fig. 3a) [40]. Recently, it has been confirmed that ellagic acid can significantly inhibit CYP3A and P-glycoprotein (P-gp)-mediated efflux in the intestine [41], thus, increase warfarin plasma concentration and its pharmacological effect.

As for the pharmacokinetic interaction of warfarin with guava leaves extract and quercetin, and the effect both exerted on increasing warfarin C_max_, the interaction is possibly accounted to different reasons including; the availability of flavonoids in guava leaves, including quercetin, which had a very strong affinity for the same primary binding site of warfarin on serum albumin. Consequently, their simultaneous uptake may cause a competitive inhibition by displacing warfarin, leading to an increase in its free form, that would increase warfarin plasma concentration [22, 36]. Another reason can be attributed to quercetin which exerts absorption enhancement activity by inhibiting the intestinal efflux pumps [42]. Absorption enhancement might cause increase in the warfarin’s plasma C_max_ and AUC. Furthermore, the ability of guava leaves extract and quercetin to inhibit some of hepatic CYP isoforms associated with warfarin metabolism such as CYP2C9, 2C8 and 3A4 may be responsible for increasing warfarin plasma concentration [40, 43]. No previous studies have reported the pharmacokinetics of warfarin in presence of guava leaves extract, pomegranate peel extract, quercetin or ellagic acid.

In vitro warfarin-herb interactions and their effects on hepatic CYP isoforms showed that CYP2C9, 2C8 and 3A4 activities in primary isolated and cultured rat hepatocytes were not affected by warfarin (Figs. 3a, 4a and 5a).

Pomegranate peel, did not have any significant effect on the three CYPs in rat hepatocytes whether used alone or in combination with warfarin (Figs. 3a, 4a and 5a). Since CYP isoforms other than 2C9, 2C8 and 3A4 are involved in warfarin metabolism, further study with more focus on pomegranate peel and its constituents is therefore suggested.

Significant reduction exerted by the use of ellagic acid whether used alone or concomitantly with warfarin is in line with those reported in previous studies [40] which showed that ellagic acid had inhibitory effect on many CYPs isoforms including CYP2C9. It is generally known that CYPs often possess enzyme specific catalytic repertoires and can display exquisite catalytic selectivity for regio- and stereo-specific reactions [44]. The mammalian isoforms have been reported to attain some degree of flexibility in its structure, including what is called peripheral binding sites that can influence substrate binding and oxidation. Therefore, it can be suggested that ellagic acid may affect warfarin binding to CYP2C8 and CYP3A4 by changing the conformation of these enzymes that leads to more affinity towards warfarin binding [45, 46]. These results contradict those of a previous study by Kaneko, 2013 where ellagic acid had a clear inhibitory effect on CYP2C8 and CYP3A4 activities [40]. In reviewing the literature, no data was found on warfarin combinations with pomegranate peel extract and ellagic acid effect on CYP2C9, 2C8 and 3A4 activities.

The observed reduction in CYP2C9 activity upon treatment with by guava leaves extract, quercetin and their combinations with warfarin could be attributed to the competitive inhibitory effect of quercetin on CYP2C9 [37]. These results were consistent with previous studies that demonstrated the inhibitory effects of guava leaves extract and quercetin on CYP2C9 activity [40, 43].

Although guava leaves extract showed an insignificant effect on CYP2C8 when used alone, it significantly reduced its activity when combined with warfarin (*p < 0.01*) (Fig. 4b). In a previous study conducted by Kaneko in 2013, it was reported that guava leaves extract had an inhibitory effect on CYP2C8 [40]. Therefore, it might be suggested that CYP2C8 enzyme can be influenced by the presence of warfarin, which can modify the uncompetitive binding of the active extract components on the enzyme. It seems possible that some competition could have happened between quercetin and warfarin on the binding site of CYP2C8 since previous studies showed that quercetin can competitively inhibit the effect of CYP2C8 [40, 47]. Thus, explains the influence of quercetin in reducing CYP2C8 activity in tested rat hepatocytes.

It has been suggested that guava leaves extract constituents, such as gallic acid and ursolic acid, have the ability to diminish the activity of CYP3A4 in human liver microsomes [37, 48]. That comes in line with the results obtained from testing guava leaves extract both alone and in combination with warfarin in significantly affecting CYP3A4 activity in primary isolated rat hepatocytes. Further reduction in CYP3A4 activity was observed upon administerating quercetin concomitantly with warfarin, suggesting that CYP3A4 may undergo an allosteric change during its binding with warfarin that possibly enhances quercetin binding which leads to increase its inhibitory effect [49]. Such assumption needs further investigation.

Finally, it was clear that guava leaves extract, quercetin and ellagic acid showed pharmacodynamic and pharmacokinetic interactions with warfarin. The possible mechanism for that is their inhibitory effect on CYP2C9, 2C8 and 3A4 isoforms which are responsible for warfarin metabolism. Conversely, pomegranate peel extract only showed pharmacodynamic interaction when combined with warfarin as it did not affect pharmacokinetic parameters or CYPs activity. It seems other mechanisms involved in the demonstrated pharmacodynamic interaction of pomegranate peel extract.

## Conclusion

All studied combinations enhanced the anticoagulant effect of warfarin. The interaction between warfarin and pomegranate peel was mainly pharmacodynamic. On the other hand, ellagic acid, guava leaves and quercetin exhibited both pharmacodynamic and pharmacokinetic interactions. The previously mentioned interactions might be attributed to the inhibitory effects of guava leaves, quercetin and ellagic acid on CYP2C9, 2C8 and 3A4 that are responsible for the metabolism of warfarin.

The current investigation suggests a high risk of bleeding that may arise from the co-administration of pomegranate peel and guava leaves extracts with warfarin therapy. It should always be considered that other active components might be present in the tested plants in addition to the presence of other isoforms of CYPs involved in warfarin’s metabolism and all these components is suggested to be investigated in the future. Currently however, awareness on the reported interactions is essential to be spread among the healthcare practitioners and should be taken it into consideration in order to minimize any unnecessary interactions between warfarin and the reported herbal constituents.

## Abbreviations

AUC: Area under curve
AUMC: Area under moment curve
Ca: Calcium
CL: Clearance
Cmax: Maximum plasma concentration
CMC: Carboxy Methyl Cellulose
CV: Coefficient of Variation
CYP: Cytochrome
DMEM: Dulbecco’s Modified Eagle Medium
DMSO: Dimethyl Sulfoxide
EA: Ellagic acid
EDTA: Ethylene Diamine Tetra Acetic Acid
EMEA: European Medicines Agency
FELASA: Federation European of Laboratory Animal Sciences Association
GvL: Guava leaves Extract
HBSS: Hank’s Balanced Salt Solution
HEPES: (4-(2-hydroxyethyl)-1-piperazineethanesulfonic acid)
HPLC: High Performance Liquid Chromatography
HPV: Hepatic Portal Vein
INR: International Normalized Ratio
IS: Internal Standard
ISI: International Sensitivity Index
IVC: Inferior Vena Cava
Kel: Elimination rate Constance
Mg: Magnesium
MRT: Mean Residence Time
PK: Pharmacokinetic
PPE: Pomegranate Peel Extract
PT: Prothrombin Time
QC: Quality Control
Que.: Quercetin
R2: Regression
RPM: Rotation Per Minutes
SD: Standard Deviation
SEM: Standard Error of Mean
t0.5: Half Life
Tmax: The time in which maximum plasma concentration is reached
Vz: Volume of Distribution
WF: Warfarin
